# Supplementary material for: Parental developmental screening with CARE: A pilot hybrid assessment and intervention with vulnerable families in Colombia
Source: PLoS One. 2023 Jun 28;18(6):e0287186. doi: 10.1371/journal.pone.0287186 (PMC10306226; doi:10.1371/journal.pone.0287186)
Supplement: Attachment — Submitted filename: Response to Reviewers_PONE_2022.docx [file pone.0287186.s002.docx]

PONE-D-21-40928

Parental developmental screening with CARE: a remote assessment and intervention with vulnerable Spanish-speaking families

PLOS ONE

Dear Editors

About before reviewers’ comments and observations, following are all and every reply.

1. The reviewer has raised a number of concerns that you should respond to as part of your revisions. Please ensure that you provide further detail on the intervention not reported here and why the results of this have not been reported.

Reply/action: information is extended in pages 11-12 for major clarity.

Participants for the not reported intervention had no information or effect on the completed CARE booklet intervention (CBI) or the results from the intervention development process (Duncan et al., 2020). The not reported intervention and the quasi-RCT protocol help to prevent the heterogeneity of treatment effect (Averitt et al., 2020), using randomized blocks of six with a computer-generated number sequence that was created a priori on the random.org website. The reason for not reporting the excluded intervention is limitations for feasible and comparative analysis with the CBI due to the sample size and the agreement with the independent founding agency for the other intervention to obtain a significant number of participants before publications, including their data. An independent research assistant informed participants of their group allocation. The 48 families were randomized to one of two conditions (not reported intervention, *n* = 25; CARE intervention group-only, *n* = 23), and the remaining 27 (those who did not attend the allocation meeting) were assigned to the control group. We cannot establish if they did not attend the allocation session should affect our results. Consequently, we assumed the main reason they did not attend was that they were not available on the meeting date, and there is no reason to assume that any material features defining this group would bear on child outcome. The lack of demographic differences between the groups in the final sample (*N* = 50) supports this assumption.

2. In addition to the reviewer’s requests, please clarify the aims of the study in your Introduction. The work is noted as a pilot study – please outline the specific research questions you aimed to address by performing the work.

Reply/action: information is extended in pages 5-6 for major clarity about the end of introduction, before the Method section.

Early developmental screening (EDS) with well-designed tools has been deployed to assess the effect of intervention programmes on specific outcomes (Fernald, Kariger, Engle & Raikes, 2009; Tann et al., 2021). Well-designed EDS tools also reduce financial and time costs for fundamental research and public health activities, such as assessing early developmental status at an individual level (Johnson, Wolke, & Marlow, 2008), even in LMICs (Tann et al., 2021).

In the last decade, various studies have evaluated EDS tools deployed at primary healthcare services in LMICs (Fischer, Morris & Martines, 2014; Fernald, Prado, Kariger & Raikes, 2017; Boggs et al., 2019). The mentioned review studies did not find any screening tool particularly designed for or used in Colombia (Boggs et al., 2019; Fischer et al., 2014). However, Colombia’s Ministry of Health uses the Abbreviated Development Scale (ADS-1; in Spanish, Escala Abreviada del Desarrollo; Ortiz, 1991) in various institutional scenarios, including children’s centres and public kindergartens around the country. Colombia’s Ministry of Health (Ministerio de Salud, 2016) presents the ADS-1 with no published report on its conceptualization, pilot testing, or complete analysis of validity and reliability. A partial validation analysis of the ADS-1 for the language and hearing domain in 4- to 5-year-old children indicated low predictive ability (Sensitivity: 54%, Specificity: 42%) and poor agreement with a gold standard for early detection of language and hearing disorders (the Reynell norm-referenced test) on measuring expressive and receptive language skills (Muñoz-Caicedo, Zapata-Ossa, & Pérez-Tenorio, 2013). We can therefore conclude that to the best of our knowledge, ADS-1 is not a very suitable tool for the Colombian context, following the standards of Boggs et al. (2019). Moreover, there is no interventions or studies about the “administration of test” effects, as recommended by several researchers (e.g., Boggs et al., 2019; Fernald et al, 2017). The “administration of test” view requires comparing caregiver reports with direct child observation. Vitrikas et al. (2017) described both a parent-completed EDS tool as an instrument for obtaining screening information through parent participation, and (as a separate instrument) a directly administered EDS tool when information is based on direct observation of the child by a physician or other expert. To probe the potential of a EDS tool to improve the outlook of at-risk children in conditions of poverty in LMICs, the current study describe the design of a parent-administered report of direct observation and activities for children between 36 and 59 months of age. More specifically, it presents the further development of a direct assessment tool conducted by parents and other caregivers called CARE (i.e., Compilation of Activities to Report and Enhance development). We define “parent report” as information obtained from a parent using CARE. The idea for the design of CARE is associated with the truncated continuity of a research program in Colombia, the Inicio Parejo de la Vida (IPV; “Equal Start in Life”, in English).

Also, the hypothesis expressed in the original manuscript change to a question in the page 10:

We will answer the following research question for a pilot design: Would the CARE booklet intervention (CBI) with parents from vulnerable and scarcity-conditions neighborhoods in Colombia benefit their children’s cognitive development and language abilities? As a pilot design, it is necessary to note the exploratory conditions for the current study and the consequent precaution in the analysis of the results.

3. A significant limitation of the work is the assignment of the control condition. Please mention this concern in your Abstract. Please also note your work as a pilot study in your Title.

Reply/action: all corrections made it and Included the explanatory phrase for the pilot Quasi-Randomised Control Trial design:

(i.e.,Control group participants assigned based on non-random criteria)

4. When submitting your revision, we need you to address these additional requirements.

2. We note that the grant information you provided in the ‘Funding Information’ and ‘Financial Disclosure’ sections do not match.

When you resubmit, please ensure that you provide the correct grant numbers for the awards you received for your study in the ‘Funding Information’ section.

Reply/action:

This work was supported the corresponding author dedication by grants awarded by the internal research fund of the Universidad de la Sabana (https://www.unisabana.edu.co/investigacion/fomento-de-la-investigacion/) and, Ministerio de Ciencia, Tecnología e Innovación de Colombia – Minciencias (https://minciencias.gov.co/formacion-de-alto-nivel/apoyo-nacionales-exterior), Grant #860, for Doctoral studies support. Opinions, findings, and conclusions from this report are those of the authors and do not necessarily reflect the views of Minciencias. The funders had no role in study design, data collection and analysis, decision to publish, or preparation of the manuscript.

3. We note that you have indicated that data from this study are available upon request. PLOS only allows data to be available upon request if there are legal or ethical restrictions on sharing data publicly. For more information on unacceptable data access restrictions, please see http://journals.plos.org/plosone/s/data-availability#loc-unacceptable-data-access-restrictions.

In your revised cover letter, please address the following prompts:

a) If there are ethical or legal restrictions on sharing a de-identified data set, please explain them in detail (e.g., data contain potentially sensitive information, data are owned by a third-party organization, etc.) and who has imposed them (e.g., an ethics committee). Please also provide contact information for a data access committee, ethics committee, or other institutional body to which data requests may be sent.

Reply/action:

Under family confidentiality agreement and consent before and during the COVID-19 pandemic and the legal restrictions from the recruitment protocols followed by the pre-schools and daycare centres located in Colombia, the raw data supporting the results and conclusions of this study are available on request from the corresponding author, without undue reservation, after the direct allowance of the institutional committees at Universidad de la Sabana.

4. Please include your full ethics statement in the ‘Methods’ section of your manuscript file. In your statement, please include the full name of the IRB or ethics committee who approved or waived your study, as well as whether or not you obtained informed written or verbal consent. If consent was waived for your study, please include this information in your statement as well. 

Reply/action: Included in page 12 of the manuscript.

The study and the intervention involving human participants were reviewed and approved by the board at the Faculty of Psychology (Facultad de Psicología). The General Directorate of Research (Dirección General de Investigaciones) of the Universidad de la Sabana also granted ethical approval for the study (Acta CAG #1517 of 19/11/2015). Permission for data collection was granted in agreement with the legal ruling of Resolution No 008430 of 1993 of the Ministerio de Salud de la República de Colombia (Health Ministry of Colombia), which sets out ethical, scientific, technical and administrative norms for research activity with human participants. Written informed consent from the participant's legal guardian/next of kin was required to participate in this study following the mentioned national legislation and the institutional requirements.

Reviewers' comments:

Reviewer's Responses to Questions

**Comments to the Author**

1. Is the manuscript technically sound, and do the data support the conclusions?

The manuscript must describe a technically sound piece of scientific research with data that supports the conclusions. Experiments must have been conducted rigorously, with appropriate controls, replication, and sample sizes. The conclusions must be drawn appropriately based on the data presented.

Reviewer #1: Yes

2. Has the statistical analysis been performed appropriately and rigorously?

Reviewer #1: Yes

3. Have the authors made all data underlying the findings in their manuscript fully available?

The [PLOS Data policy](https://nam02.safelinks.protection.outlook.com/?url=http%3A%2F%2Fwww.plosone.org%2Fstatic%2Fpolicies.action%23sharing&data=05%7C01%7Cjuan.giraldo9%40unisabana.edu.co%7C780936b78e844d6fd37d08da6ff2bf3d%7Caca5163100fe490d91ab163ef87260ee%7C0%7C0%7C637945385796990821%7CUnknown%7CTWFpbGZsb3d8eyJWIjoiMC4wLjAwMDAiLCJQIjoiV2luMzIiLCJBTiI6Ik1haWwiLCJXVCI6Mn0%3D%7C3000%7C%7C%7C&sdata=1Y9MjTAA1ogS7htbgO1ZEBKjwp%2FZhn4zuRCM2Wp0nhw%3D&reserved=0) requires authors to make all data underlying the findings described in their manuscript fully available without restriction, with rare exception (please refer to the Data Availability Statement in the manuscript PDF file). The data should be provided as part of the manuscript or its supporting information, or deposited to a public repository. For example, in addition to summary statistics, the data points behind means, medians and variance measures should be available. If there are restrictions on publicly sharing data—e.g. participant privacy or use of data from a third party—those must be specified.

Reviewer #1: No

4. Is the manuscript presented in an intelligible fashion and written in standard English?

PLOS ONE does not copyedit accepted manuscripts, so the language in submitted articles must be clear, correct, and unambiguous. Any typographical or grammatical errors should be corrected at revision, so please note any specific errors here.

Reviewer #1: No

Reply/action: A revision was made before resubmission by a native English speaker, a highly qualified University associated professor living in Colombia.

5. Review Comments to the Author

Please use the space provided to explain your answers to the questions above. You may also include additional comments for the author, including concerns about dual publication, research ethics, or publication ethics. (Please upload your review as an attachment if it exceeds 20,000 characters)

Reviewer #1: In this manuscript, the authors investigate findings from a pilot quasi-randomized controlled trial of a parenting intervention that combined parent education sessions regarding activities associated with developmental stimulation, and parental monitoring through a home screening booklet (CARE) among families of low socioeconomic status in a LMIC. They found that children whose caregivers were assigned to the intervention screened for fewer developmental delays post intervention than children in the control group. They also found interactions in effects with child gender, maternal education, and family socioeconomic status.
These findings are of potential interest, are important for continued research in child development in LMIC, and provide support for a larger study of this intervention.

However, I have several concerns about the paper, which I have outlined below:

Introduction
1. In the Introduction, the authors note that “only a limited number of studies have examined the impact of early parenting interventions on the cognitive performance of children living in poverty; and most of them have reported only limited data about early learning or intervention conditions.” The authors should include studies by Mendelsohn and colleagues of a reading aloud intervention in Brazil that addresses these issues:

Weisleder, A., Mazzuchelli, D. S., Lopez, A. S., Neto, W. D., Cates, C. B., Gonçalves, H. A., ... & Mendelsohn, A. L. (2018). Reading aloud and child development: a cluster-randomized trial in Brazil. Pediatrics, 141(1).

Mendelsohn, A. L., da Rosa Piccolo, L., Oliveira, J. B. A., Mazzuchelli, D. S., Lopez, A. S., Cates, C. B., & Weisleder, A. (2020). RCT of a reading aloud intervention in Brazil: Do impacts differ depending on parent literacy?. Early Childhood Research Quarterly, 53, 601-611.

Reply/action: Included in page 8 of the manuscript

Brazil is the only South American country with an early parenting interventions intervention interested in children’s the cognitive performance (Weisleder et al., 2018). The early intervention reported in Brazil is a parent-child programme (Weisleder et al., 2018) followed the *Reach Out and Read* and the *Video Interaction Project* (Mendelsohn et al., 2018, 2020) with strategies for families under intervention, borrowing children’s books on a weekly basis and in focusing on reading aloud in monthly parent workshops. Weisleder et al. (2018) found that parents in the intervention group engaged in significantly greater cognitive stimulation (*d* = 0.43) and higher quantity and quality of reading interactions (*d* = 0.52–0.57) than controls. Also, at a 9-month follow up, children in the intervention group scored significantly higher than controls on receptive vocabulary (*d* = 0.33), working memory (*d* = 0.46), and IQ (*d* = 0.33).

Methods
2. Consistency in the name of the intervention would be helpful. Although the authors call the intervention CBI throughout most of the manuscript, they return to CARE several times (e.g., pp. 10, para. 2).

Reply/action: Included in several pages where inconsistencies was detected.

3. The authors indicate that there was another intervention that was not included in the present analyses, but use the acronym for that intervention in the manuscript (DBS; pps. 11 & 25). Naming this intervention from the outset, with a brief description and indication of why it is not included in the present analyses would be a better way to handle this.

Reply/action: as mentioned before, information is extended in pages 11-12 for major clarity.

Participants for the not reported intervention had no information or effect on the completed CARE booklet intervention (CBI) or the results from the intervention development process (Duncan et al., 2020). The not reported intervention and the quasi-RCT protocol help to prevent the heterogeneity of treatment effect (Averitt et al., 2020), using randomized blocks of six with a computer-generated number sequence that was created a priori on the random.org website. The reason for not reporting the excluded intervention is limitations for feasible and comparative analysis with the CBI due to the sample size and the agreement with the independent founding agency for the other intervention to obtain a significant number of participants before publications, including their data. An independent research assistant informed participants of their group allocation. The 48 families were randomized to one of two conditions (not reported intervention, *n* = 25; CARE intervention group-only, *n* = 23), and the remaining 27 (those who did not attend the allocation meeting) were assigned to the control group. We cannot establish if they did not attend the allocation session should affect our results. Consequently, we assumed the main reason they did not attend was that they were not available on the meeting date, and there is no reason to assume that any material features defining this group would bear on child outcome. The lack of demographic differences between the groups in the final sample (*N* = 50) supports this assumption.

4. The manuscript indicates that CBI was a remote intervention, but this was not discussed in the description of the program in the Methods section. Method of delivery should be included there.

Reply/action: information is extended in pages 13-14 for major clarity.

According to the participant information and preliminary data analysis (see Table 1), all participants fell under the definition of multidimensional poverty (Aguilar & Sumner, 2020). This means that they had low socioeconomic status, with multiple factors affecting them including lower-than-average income on a national scale, health or nutritional deficiency, lack of continuous attendance at nursery or preschool services, and material scarcity in some standards of living (e.g., essential home utilities). Initial analysis using the Mann-Whitney test (Supplementary Table 1) showed no significant differences between groups in various sociodemographic factors (i.e, children’s age in months, mother’s education level, reading engagement, mother’s employment, and socioeconomic status). CBI participants were asked to have at least an active phone number, a chat app or an email address, and to complete an intervention session each week for six weeks. Also, the participants promised to review the intervention materials and answer some questions via phone, chat or email. Assessments were conducted at a local Children’s Centre (CC) for baseline and again not more than two weeks after the last session. Even though face-to-face assessment was conducted at the CC, the CBI is considered a remote intervention with a mix of moderated (i.e., the engagement monitoring via phone call, chat or email message) and unmoderated procedures (i.e., the delivered CARE booklet) to obtain and deliver related information (Amso, Cusack, Oakes, Tsuji, & Kirkham, 2022).

5. It would strengthen the manuscript to include comparison statistics between the CBI and Control groups (t-tests or chi-squares) on the descriptive statistics in Table 1. This would provide further evidence that the groups are comparable, even though assignment was done through a quasi-random procedure.

Reply/action: recommendation included in page 14.

Initial analysis using the Mann-Whitney test (Supplementary Table 1) showed no significant differences between groups in various sociodemographic factors (i.e, children’s age in months, mother’s education level, reading engagement, mother’s employment, and socioeconomic status).

Results
6. The results section would benefit from additional details about the interactions found in the two-way ANCOVAs. Were effects greater for girls vs. boys, mothers with higher vs. lower education, higher vs. lower SES? This would provide important information for planning a larger study.

Reply/action: recommendation implied an adjustment in p. 21 with two more technical references:

Due to the small sample, as a first step in analysis, we conducted two-way ANCOVAs for a parametric check of interactions between factors (i.e., CBI vs Control) and sociodemographic variables (i.e., child age, gender, principal caregiver’s education level, reported reading engagement at home, mother’s employment status, and socioeconomic stratification) controlled by pre-intervention scores on each assessment. Then, after the two-way ANCOVA procedure, the interaction (i.e., group * sociodemographic variable) was removed, and the ANCOVA (i.e., one way) was rerun with only main effects for intervention groups and pre-intervention scores, including all the measurements (i.e., primary and secondary outcomes) and the Cautions and Delay items in the four developmental domains observed in the primary outcome measurements. The unequal sample size in each group prevents the exhaustive pairwise analysis to complete the two-way and one-way ANCOVAs, including post-hoc procedures, like the *Scheffé’s* test, to find out which pairs of means are significant (Westfall, 1997; Westfall & Young, 1993). Instead of the exhaustive pairwise analysis, the effect size is reported following the principles and recommendations of Lakens (2013) and Pek & Flora (2018) for eta-squared (η^2^) and partial eta-squared (η^2^_p_) interpretation, that express the amount of variance accounted for by one or more independent variables. The following rules of thumb are used to interpret values for η^2^ and η^2^_p_: .01= small effect size, .06 = medium effect size, .14 or higher = large effect size

7. I would also consider presenting the two-way ANCOVA findings after the main effects findings from the one-way ANCOVA, since the main effects are the primary question of interest.

Reply/action: we follow the recommendation with the clarifying adjustment in p. 21 described before.

8. The results may be easier to follow if primary and secondary outcomes were separated, with results for the dimension so the HLL screening included with the primary outcome findings.

Reply/action: we follow the recommendation with the following adjustments in p. 25 and 26.

## **Primary outcomes comparison in pre-post assessments for CBI and Control Group**

One-way ANCOVAs were conducted to compare the effectiveness of the CARE intervention (CBI) in the post-test measurements whilst controlling for pre-test measurements values. Levene’s test and normality checks were carried out and the assumptions were met. There was a significant difference between the CBI and control groups on the HLL Delay items, *F*(1, 47) = 10.45, *p* = .002, partial η^2^ = .182. No differences were found in other primary measurements when CBI and control groups were compared. ANCOVA results in CBI vs Control comparison indicate that mean post-tested Delay ítems in HLL observations differed between the parental training conditions and the control group, with a positive association between the pre-test and post-test counts of Delays. Children in CBI conditions displayed adjusted post-test Delays means that were higher than the mean for the control group. Fig 3 below shows the significant nature of the association for the CBI compared to the Control group.

## **Secondary outcomes comparison in pre-post assessments for CBI and Control Group**

One-way ANCOVAs were conducted to compare the effectiveness of the CARE intervention (CBI) in the post-test measurements whilst controlling for pre-test measurements values. Levene’s test and normality checks were carried out and the assumptions were met. There was a significant difference between the CBI and control groups on the Frog Story’s narrative devices scores, F(1, 17) = 4.87, p = .041, partial η^2^ = .223. No differences were found in other secondary measurements when CBI and control groups were compared.

9. Figure 3 seems to indicate that children with more pre-test delays also had more post-test delays in the CBI group, but not in the Control group. Given that delays increased in the Control, but not the CBI, group, interpretation of this is difficult. Does this indicate that additional children developed delays in the Control group? A different figure may better illustrate the point of the analysis.

- Reply/action: we follow the recommendation with a new Figure with R (ggplot2) and the following adjustments as Note in p. 26:

*Note*. This Figure demonstrates a positive effect of the CBI intervention in diminishing the post Delays. The solid lines in colour represent the regression slopes for the CBI (Violet) group. The black line corresponds with the regression slope in the Control group and indicates a negative relation for the pre and post Delays. The grey shadow areas for each line represent the standard error for each trendline data.

Discussion
10. The discussion of parent engagement is interesting, but seems out of place in the limitations section. The manuscript would be stronger if this was included in the Introduction and general Discussion, along with a limitation noting the possibly low rate of up-take and inability to measure what parents actually did at home, since written and verbal indicators did not match.

Reply/action: we follow the recommendation with the following changes:

In Introduction, p. 9-11:

**The CARE Booklet-Intervention (CBI)**

CARE acts as a guide for parents on how they can assess and track their child’s developmental progress, and how they can provide scaffolding activities to promote their child’s development. However, the strict delivery of any screening tool for parent administration, like CARE, is not sufficient as an intervention. A structured that combined the opportunity to measure children in scenarios like children centres for low-income families in Colombia and remote protocols for register parent-child interactions at home, helps to give structure to the CARE booklet-intervention (CBI). The CARE booklet assessing children’s characteristics is like home-based records (HBRs). An HBR such CARE regular and frequently running in parallel with a CBI intervention for optimal health and educational systems is not only desirable (Mahadevan & Broaddus-Shea, 2020): it could increase confidence and engagement in parental activities at home. The parental engagement for daily activities at home are related in higher order thinking about their own behaviour and how it can enhance, for example, their child’s vocabulary development (Teepe et al., 2019).

Also, the CBI could work well at home when interactions might be a parental task or burden. Constant recall and demand for interactions at home might increase parental stress and the possibility of parental burnout. Recent studies in the prevalence of parental burnout (Roskam et al., 2021) show that cultural and social values like individualism and number of hours spent paying attention to children play a significant role in many LMICs, including Colombia. Specific societal values also play a role in Roskam and colleagues’ research. Colombia had the highest scores on the “Indulgence” scale, meaning, according to the researchers, that participants (*N* = 95) allow relatively free gratification of basic and natural human drives related to enjoying life and having fun. CBI might transform parental “demand” for interactions with their children into activities reported in the booklet, not for evaluation of parenting, but intended as a support device. Parent support in CBI messages had three principal interpretations: active learning is central to individual child development; social interactions are fundamental to caregiver-child engagement (Haine-Schlagel & Escobar-Walsh, 2015); and (paraphrasing Lave, 1988) development is not an independent process among other processes but, like learning, it is an aspect of any interactive process in the world.

In the CBI, the relevance of parental screening it is not a matter of how much children change and develop but a matter of how, when and with whom they interact. The child might develop what they were not expected to report and might not perform according to the kind of development they were expected to show. However, before any hypothesis about indulgence and parental stress or engagement (Haine-Schlagel & Escobar-Walsh, 2015), is necessary to know if the CBI and mediating sociodemographic factors might change children’s outcomes, as other parental interventions has been shown to do (Biel et al., 2020).

In the discussion, p. 31-32:

The microsystems dynamics related with the CBI had focused on promoting engagement with sensitive and responsible parenting, but indicated distinct barriers (e.g., employment challenges, health-related challenges) and facilitators (e.g., knowing other mothers in the group, interest in the program topics) had a significant effect in recent studies with socioeconomically disadvantaged families (So et al., 2020), none of which were explored in the current CBI study. Also, CBI is an intervention for child development supported by current models of early childhood interventions recommended in scalable programs to impact early development (Cavallera et al., 2019; Pérez-Escamilla, Cavallera, Tomlinson & Dua, 2018), specifically in dedicated caregiving from parents (Martins et al., 2020) and low-cost interventions, contrary to exclusively use home visits. The emphasis in remote CBI compared with exclusively home visits interventions is not only monetary: high parental engagement with home visits is associated with particular maternal characteristics (higher IQ, older mothers, mothers who were employed during pregnancy, mothers with greater knowledge of infant development, and mothers with more positive parenting beliefs), rather than other characteristics (i.e., young, unemployed, and/or less well-educated mothers) that are more frequently found in LMICs (Doyle, 2020).

Overall
11. A minor concern relates to clarity and grammaticality throughout the manuscript. The article would benefit from a thorough review.

Reply/action: A revision was made before resubmission by a native English speaker, a highly qualified University associated professor living in Colombia.

6. PLOS authors have the option to publish the peer review history of their article ([what does this mean?](https://nam02.safelinks.protection.outlook.com/?url=https%3A%2F%2Fjournals.plos.org%2Fplosone%2Fs%2Feditorial-and-peer-review-process%23loc-peer-review-history&data=05%7C01%7Cjuan.giraldo9%40unisabana.edu.co%7C780936b78e844d6fd37d08da6ff2bf3d%7Caca5163100fe490d91ab163ef87260ee%7C0%7C0%7C637945385796990821%7CUnknown%7CTWFpbGZsb3d8eyJWIjoiMC4wLjAwMDAiLCJQIjoiV2luMzIiLCJBTiI6Ik1haWwiLCJXVCI6Mn0%3D%7C3000%7C%7C%7C&sdata=xX%2Bz%2BW7Bx0aDlPUJvC7m%2FPt2mjFW2YwSiQtifYBNy3w%3D&reserved=0)). If published, this will include your full peer review and any attached files.


If you choose “no”, your identity will remain anonymous but your review may still be made public.


**Do you want your identity to be public for this peer review?** For information about this choice, including consent withdrawal, please see our [Privacy Policy](https://nam02.safelinks.protection.outlook.com/?url=https%3A%2F%2Fwww.plos.org%2Fprivacy-policy&data=05%7C01%7Cjuan.giraldo9%40unisabana.edu.co%7C780936b78e844d6fd37d08da6ff2bf3d%7Caca5163100fe490d91ab163ef87260ee%7C0%7C0%7C637945385797147481%7CUnknown%7CTWFpbGZsb3d8eyJWIjoiMC4wLjAwMDAiLCJQIjoiV2luMzIiLCJBTiI6Ik1haWwiLCJXVCI6Mn0%3D%7C3000%7C%7C%7C&sdata=Ru600NvCLfwH5zEsoYvHLkghXM0ULfEu%2F7Ah%2BP2VXjo%3D&reserved=0).

Reviewer #1: No
