# Supplementary material for: Parental developmental screening with CARE: A pilot hybrid assessment and intervention with vulnerable families in Colombia
Source: PLoS One. 2023 Jun 28;18(6):e0287186. doi: 10.1371/journal.pone.0287186 (PMC10306226; doi:10.1371/journal.pone.0287186)
Supplement: Attachment — Submitted filename: Response to Reviewers_PONE-D-21-40928R1.docx [file pone.0287186.s003.docx]

PONE-D-21-40928R1

Parental developmental screening with CARE: A pilot remote assessment and intervention with vulnerable families in Colombia

PLOS ONE

Dear Reviewers, I am very grateful for the opportunity to respond to your review of my manuscript. The full replies are below.

Best regards,

Juan J Giraldo-Huertas, PhD

To Major Comments: 1. In all, clarification of which parts of the intervention require internet access, which require in person appearances, and which are entirely remote, is needed. The authors state that remote interventions are needed in order to allow wider access (i.e., access for those not able to attend in person and/or without internet or phone access). However, it appears that the present intervention requires some phone or internet access (e.g., email) as well as some in-person sessions (e.g., intervention meetings and parent small group meetings). Explicit clarification on format for different parts of the intervention is needed. Given multiple formats utilized, the intervention may be better portrayed as hybrid, than fully remote.

R/ **more precision in the content of the Method Section and specifical indications about the modality of participation are included in Table 2 (p. 18).**

To Minor Comments:
1. Given the study’s focus on the benefits of intervention, it is recommended the study is referred to as an efficacy study as opposed to a validation study.

R/ **Corrected in abstract (p. 2)**

2. In the introduction the authors note “The selection criteria for the HLL are intended to increase the rigor in items for observation in Latin American Spanish…” Is the goal to state that this is the reason the authors have selected this measure, or that this is the rationale for the measures development? Please clarify?

R/**Corrected in p. 7:**

**The selection criteria for the HLL serve two purposes. Firstly, they are intended to allow for easy and rigorous adaptation of developmental screening items in Latin American Spanish through cultural and linguistic modifications obtained from the original Llevant study in the Basque Country (Fuentes-Biggi, Fernandez, & Alvarez, 1992). Secondly, the HLL is a widely-used and standardized adaptation of the DDST in various countries, including densely populated areas in Brazil (Lopez-Boo, Cubides-Mateus, & Llonch-Sabatés, 2020) and Colombia (Rubio-Codina & Grantham-McGregor, 2020).**

3. Authors state “Results of a receiver operating curve (ROC) analysis indicated that parental report using CARE is a satisfactory tool for screening diagnostics (Area Under the Curve - AUC: 0.894, trapezoidal Wilcoxon area)..” Are these analyses done as a part of the present study? If no, please cite. If yes, please include these in results

section.

R/ **Corrected in p. 7:**

**All the reliability, agreement and diagnostic-characteristics analysis results were previously carried and published before the intervention and efficacy study (Giraldo-Huertas & Schafer, 2021).**

4. Are secondary outcomes, e.g., literacy skills, assessed in English or Spanish? This is currently not specified and clarification would be helpful.

R/ **Corrected in p. 19:**

**The outcomes were assessed exclusively in Spanish at a post-intervention assessment conducted 7-8 weeks following the baseline assessment. The assessments were conducted in-person at the Children’s Centre by two native Spanish-speaking graduate psychologists who were blind to group membership and trained in the specific measures used. The full set of assessments took 60-90 minutes. The following primary and secondary outcomes were administered at baseline and follow-up using the official or standardized version in Spanish.**

5. In the methods section, a brief discussion of reasons respondees declined participation if known, is warranted. If known, please also discuss how this may have impacted results in the discussion section.

R/ **Corrected in p.12 and p. 36.**

The baseline assessment was conducted if the child met the entry criteria and the parents consented to participate. Prior to the COVID-19 pandemic (July 2019 – February 2020), the screening process identified 268 families (see Fig 1 for the CONSORT flowchart), but 156 families (54.9%) declined to enter the study and were not followed up further. The reasons for their decision were not collected.**(p. 12)**

The present study has highlighted significant practical and methodological challenges in conducting an RCT of a remote intervention for parents in low-income conditions with preschool children at risk of not reaching their developmental potential. One major obstacle in our study was the high rate of declining to join after the initial call, which affected our recruitment rate - more than half of the potential families who could have received the intervention (54.9%) opted not to participate. Unfortunately, the reasons for their decision were not collected. Recent meta-analyses and evidence-based studies (Kumar et al., 2022) report that failure to inform on attrition rates and reasons for attrition can impact the integrity of interventions in LMICs, even in home visit intervention designs (Bai, Abulitifu, & Wang, 2022). To ensure the quality of future studies, researchers should not only collect information about attrition and non-participation but also incorporate principles and practices that align with new forms of services offered in public health initiatives, such as family-focused and community-based approaches (McConkey, 2022). **(p. 36)**

**In this correction, the following references are included:**

Bai, Y., Abulitifu, R., & Wang, D. (2022). Impact of an Early Childhood Development Intervention on the Mental Health of Female Caregivers: Evidence from a Randomized Controlled Trial. *International journal of environmental research and public health*, *19*(18), 11392. https://doi.org/10.3390/ijerph191811392

Kumar, S., Narayan, S., Malo, P., Bhaskarapillai, B., Thippeswamy, H., Desai, G., & Kishore, M. (2022). A systematic review and meta-analysis of early childhood intervention programs for developmental difficulties in low-and-middle-income countries. *Asian journal of psychiatry*, *70*, 103026. https://doi.org/10.1016/j.ajp.2022.103026

McConkey R. (2022). Responding to Autism in Low and Middle Income Countries (LMIC): What to Do and What Not to Do. *Brain sciences*, *12*(11), 1475. https://doi.org/10.3390/brainsci12111475

6. Figure 1 summarizing screening flow also needs to be edited for grammar (e.g., ‘Mother get employment,’ ‘we want to received the books’).

R/ **Corrected in Figure 1: “Mother get a full-time job” and “We want to receive the books”.**

7. Page 14, gender should also be listed as a demographic factor for which no group difference was found.

R/ **Corrected in p.14**

8. It is noted on page 17 that caregivers’ accounts were frequently inconsistent. A discussion of impact of this observation on outcomes is recommended.

R/ **Corrected in p. 17 and p. 32**

At the second session (see Table 2), and each subsequent remote session, the facilitator checked via phone call, chat, or email message if that week’s daily records had been used, and if any difficulties had been encountered. It was not possible to determine any significant changes week by week and include in the results analysis the daily reporting of activities at home because caregivers’ written, and verbal accounts were frequently inconsistent in the registration area of each booklet. **(p. 17)**

However, the consistency and quality of the daily-based information recorded in the CARE booklet were problematic, which made it challenging to identify significant changes in microsystems level by time. Although this is a common issue in research that relies on self-reported data, it is important to acknowledge and address potential sources of bias or errors in the data collection process. In future studies using the CARE booklet, alternative data collection methods or strategies to improve the quality and consistency of the recorded information may prove useful. **(p. 32)**

9. Clarification of the analysis done leading to statement that “primary outcomes presented positive improvements,” page 22, should be added.

R/ **Statement corrected in p.22: “primary outcomes presented positive score changes in the CBI group”.**

10. P-values should be added to table 4. Presenting results of primary and secondary outcomes in table format, such as that of table 4, may be helpful.

R/ **Data of P-values included in Table 4 (p. 26)**

11. Limitations and future research section in particular should be edited for grammar and clarify (e.g., sentence: A formal comparison between our parent-based intervention and a center-based intervention like that reported by Nores et al. requires a comprehensive analysis of the scientific literature relating to comparisons of interventions in Spanish-speaking and LMIC conditions; and so far, there has been no such study).

R/ **Corrected from p.33 to p.37**

All effect sizes in a pilot study should be interpreted with caution because there are several limitations due to the small sample size. A larger sample size is needed for a rigorous and detailed mapping of the effects of similar procedures on poor, low-income families in LMICs, including adequate controls for sociodemographic variation for future post hoc analysis after the two-way ANCOVA procedures to properly evaluate the CBI.

Comparing the CBI with Colombian center-based interventions like the aeioTu program (Nores et al., 2019) might not be appropriate because it requires a control group with no local service allocation. However, the CBI is a suitable alternative to consider when comparing the child-to-adult dyadic ratio and the length of the intervention. To make a formal comparison between our parent-based intervention and a center-based intervention like that reported by Nores et al., a comprehensive analysis of the scientific literature relating to interventions in Spanish-speaking and LMIC conditions is necessary, but no such study has been conducted so far.

Families allocated to the control (local services only) group were assigned to the control condition because they did not present themselves on the day of randomized allocation and cannot be considered a truly random control group. However, this reflects a real-life clinical and research situation. Future RCT studies should be completely coherent with randomized allocation and sufficient sample size to avoid potential biases and to increase statistical power to generalize the differences between groups detected.

Another limitation of our study is related to our measure of development. The Haizea-Llevant observation table (HLL) is meant as a screening tool and not a diagnostic tool. A high number of Delay and Caution items in the HLL developmental dimensions should not be strictly interpreted as a delay relative to benchmarks in comparison with children of the same age. Therefore, the results were framed in terms of children being "at risk" of a loss of developmental potential, rather than as a diagnosis of a specific developmental delay.

The generalizability of our findings may be affected by using a sample from a community-based services program. Participation in the nutritional program offered in the participating childcare center was voluntary, and parents who had concerns about their child's development may have been more likely to stay in the program than parents who found their children to be on track. This could have inflated our estimates of the prevalence of risk for developmental delay. However, our sample had relatively similar proportions of various sociodemographic risk factors as the broader group of participants previously seen as at risk of developmental delay (Edmunds, 2020; Richter et al., 2019), and for these reasons, it seems reasonable to assume that our sample does not substantially overestimate the risk for developmental delay in this low-income LMIC population.

A major limitation of this study is the lack of direct observational assessment of parent skills, such as caregiver social play sensitivity, which has been shown to produce significant positive changes in infants’ cognitive and socioemotional outcomes in previous parental and language interventions (Holmberg et al., 2022; Murray et al., 2016). The absence of such assessment was due to scarce financial resources for the Q-RCT. Additionally, levels of commitment of parental involvement (CPI) were not assessed in previous stages of recruitment. A meta-analysis by Haine-Schlagel and Escobar-Walsh (2015) reported that most studies indicate how parents face environmental and personal challenges to participate actively and concluded that levels of engagement are better understood when including a consideration of parental involvement issue. Measures of CPI fall into three main categories: global participation levels, specific participation behaviors, and completion of tasks. The average completion rate for all sessions analyzed by Haine-Schlagel and Escobar-Walsh was 49%, with a range of 19% to 89%. One possible explanation for the low engagement in optional activities for the present study (54.9% at first call) could be attributed to the sociodemographic characteristics that influenced the opportunity of adherence for participants. Comparing our sample and experimental procedure with an RCT of a play-assisted intervention for children living with foster families in extreme poverty (Worku et al., 2018), two characteristics could affect the CPI in our case. First, families tended not to cancel visits in the Worku et al. intervention. In contrast, our families received all assessments by arriving at the childcare center (CC) and interventions via chat or email message at home. Second, Worku and colleagues’ (2018) intervention was conducted in a foster family program (SOS-villages), where children lived with assigned foster families and were always cared for by an SOS-village mother or “aunt”. In future, direct measures of parental behavior and competence need to be included to demonstrate objective, generalizable difficulties in CPI.

The present study has highlighted significant practical and methodological challenges in conducting an RCT of a remote intervention for parents in low-income conditions with preschool children at risk of not reaching their developmental potential. One major obstacle in our study was the high rate of declining to join after the initial call, which affected our recruitment rate - more than half of the potential families who could have received the intervention (54.9%) opted not to participate. Unfortunately, the reasons for their decision were not collected. Recent meta-analyses and evidence-based studies (Kumar et al., 2022) report that failure to inform on attrition rates and reasons for attrition can impact the integrity of interventions in LMICs, even in home visit intervention designs (Bai, Abulitifu, & Wang, 2022). To ensure the quality of future studies, researchers should not only collect information about attrition and non-participation but also incorporate principles and practices that align with new forms of services offered in public health initiatives, such as family-focused and community-based approaches (McConkey, 2022).

Moreover, future studies aiming to improve interaction skills between caregivers and children (Biel et al., 2020) may consider using a low-cost and widely applicable booklet, such as the CARE booklet intervention, to guide parental scaffolding. Our general findings on various developmental screening and language competence variables suggest the need for further work on approaches to parental training. This should include RCTs with sufficient sample size and methodological rigor to confirm and extend these tentative findings, and to more clearly demonstrate whether parent training with scaffolding guides has specific beneficial effects on relevant skills and competences of children at risk. One type of intervention includes screening or early developmental monitoring (Cavallera et al., 2019; Goldfeld & Yousafzai, 2018) to accomplish remote assessment and intervention in the most vulnerable populations of LMIC.

**In this point, the following reference is included:**

Holmberg, E., Kataja, E. L., Davis, E. P., Pajulo, M., Nolvi, S., Hakanen, H., Karlsson, L., Karlsson, H., & Korja, R. (2022). The Connection and Development of Unpredictability and Sensitivity in Maternal Care Across Early Childhood. *Frontiers in psychology*, *13*, 803047. https://doi.org/10.3389/fpsyg.2022.803047

12. The following typos are noted:
1. “Also, thr HLL was selected” (page 7) – **Corrected**.
2. “The COVID-19 pandemic had causal effects on mental health and caregiving environments (Pitchik et al., 2021), exacerbated in contexts of poverty, which increasing the risk of children not reaching their developmental potential in Colombia)” (page 31) – **Corrected**:

**The COVID-19 pandemic had causal effects on mental health and caregiving environments, particularly in contexts of poverty. These effects have been observed in Colombia and other LMICs, and have increased the risk of children not reaching their developmental potential (Pitchik et al., 2021).**

3. Missing period after (Martins et al., 2020) (page 32) – **Corrected**.
